# Biomimetic ZnO for Dye-Sensitized Solar Cells

**DOI:** 10.3390/nano10101907

**Published:** 2020-09-24

**Authors:** Javier Orozco-Messana

**Affiliations:** Instituto de Tecnología de Materiales, Universitat Politecnica de Valencia, 46022 Valencia, Spain; jaormes@cst.upv.es

**Keywords:** biomimetic ZnO, DSSCs, low-cost Photovoltaics (PVs), Building Integrated Photovoltaics (BIPVs), ceramics

## Abstract

A research study on the application of biomimetic ZnO (from eggshell membranes) as photoanodes in dye-sensitized solar cells (DSSCs) is presented. Biomimetic ZnO powder was produced and characterized. Its surface area, crystallinity, and morphology were analyzed and compared to commercial ZnO. Then, solar cells with and without dye were assembled using both the biomimetic and commercial oxides. On the dye-less cell, the oxide assumes the role of the photon absorber, while in the dye-sensitized cells, the oxide’s major function is the separation of the electron-hole pair and conduction of the electric charges formed. The characterization of the oxides showed that the biomimetic synthesis produced ZnO with a larger surface area, smaller crystallite size, and larger light absorption, possibly due to crystalline defects. SEM analysis on biomimetic ZnO revealed a tubular microstructure formed by nanocrystals, instead of the commercial powder showing spherical particles.

## 1. Introduction

The synthesis by biomimetization of eggshell membranes is a simple technique capable of producing powders of metallic oxides with hierarchical structures assembled by nanocrystals [1]. Among the metal oxides, TiO_2_ and ZnO stand out as the most studied oxides for application in dye-sensitized solar cells (DSSCs). The possibility of obtaining ZnO with characteristics such as high surface area, the presence of light-absorbing crystalline defects, and small crystallite size [2] makes the application of these materials in DSSCs promising.

Usually considered as waste, eggshell membranes (ESMs) are unique materials with potential applications in several areas. ESMs are semipermeable materials with a high surface area and a structure composed of interconnected and interlaced biopolymer fibers, which are essential for eggshell mineralization [3]. Each fiber consists of a nucleus rich in collagen and a surface rich in glycoproteins [4]. According to [5], these fibers contain amine, amide, and carboxylic groups on their surface. These functional groups can interact with precursor molecules, anchoring them to their surface where the formation of a coating may occur. These characteristics have inspired scientists to use ESMs as a bio-template in a sol-gel process to obtain fibrous structures similar to ESMs composed of various oxides.

Examples of materials synthesized using this process are ZrO_2_ [5], CaWO_4_ [6], and PdO [7]. In general, the works that used eggshell membranes for the production of nanostructured materials obtained powders with a microstructure of interlaced fibers composed of nanocrystals of approximately 5 to 30 μm. The presence of micro- and mesopores, or even structures in tube shapes, is often reported.

Dye-sensitized solar cells, also known as Grätzel cells, utilize a mesoporous film of a semiconductor, typically TiO_2_-anatase composed of nanoparticles interconnected and sintered together in order to establish electronic conduction between particles [8]. The optimization of the photoanode microstructure is vital for the high efficiency of DSSCs. Rapid electronic transport and low recombination are desired.

Typically, the thickness of this layer is about 10 μm, and the size of the nanoparticles is between 10 and 30 nm in diameter. The porosity is 50 to 60% [9]. This mesoporous layer is deposited on a conductive and transparent oxide called TCO (transparent conducting oxide), which in turn is on a glass or plastic substrate. The most commonly used substrate is fluoride-doped tin oxide (FTO)-coated glass. Thus, over the substrate, a nanocrystalline oxide layer is deposited, and a monolayer of the sensitizer is adsorbed onto the nanocrystalline oxide layer [10]. Photoexcitation of the sensitizer results in the injection of electrons into the conduction band of the oxide, leaving the sensitizer in an oxidized state. The sensitizer is restored to its initial state by an electronic transfer of the electrolyte, usually an organic solvent containing the iodide/triiodide redox system. The ions formed by the oxidation of I^-^ diffuse by a short distance (<50 μm) through the electrolyte to the cathode, which is usually coated with a thin layer of platinum that serves as a catalyst, and the regenerative cycle is completed by the reduction from I^-3^ to I^-^ [11].

With the function of supporting the dye, and as a carrier of photoexcited electrons, the photoanode must have a high surface area to ensure a high loading of dye molecules and a rapid transport of charges from the sensitizer to the counter electrode. For many years, nanostructured TiO_2_ has been subjugated as the most efficient material for DSSCs, but many recent studies show ZnO as the most promising alternative for the substitution of TiO_2_ due to its high electron mobility [12].

The crystallographic defects also play an important role in electron conduction ability and light absorption of the semiconductors. According to [13], TiO_2_/electrolyte interface defects, grain boundaries, bulk defects, and/or surface states (the energy states generated below the conduction band) impede the transport of electrons and promote undesired recombination reactions. Studies with solar cells constructed with different types of ZnO [14] showed that high concentrations of nonradioactive defects are deleterious to the photovoltaic performance of ZnO DSSCs, whereas, for radiative defects, samples displaying orange-red photoluminescent emission exhibited better performance compared with samples associated with green photoluminescent emission.

The morphology of the semiconductor particles also plays an important role in the operation of DSSCs. The use of one-dimensional structures, such as tubes, wires, and fibers, has recently attracted much attention as new morphologies for making photoactive layers in DSSCs. In addition to the improvement in electron transport, controlling the morphology of these nanomaterials can lead to a large surface area capable of absorbing larger amounts of dye [15] and increasing the unit surface photovoltaic efficiency.

## 2. Materials and Methods

### 2.1. ZnO Powder Synthesis

The biomimetic synthesis of ZnO, described in [2], was carried out using eggshell membranes as templates for the nucleation and growth of ZnO crystals. A 0.25 M solution of zinc nitrate in water was used as precursor solution. After 24 h immersed in the precursor solution, the membranes were dried at 100 °C for 24 h and calcined at 600 °C for 30 min with a 2.5°/min heating ramp. The properties of the biomimetic ZnO were evaluated in comparison with the commercial ZnO nanopowder (Sigma Aldrich 544906 < 100 nm particle size). Samples were named as shown in Table 1.

ZnO powder was synthesized by the biomimetization of eggshell membranes and characterized in comparison to commercial powders. The performances of assembled solar cells (commercial vs. biomimetic ZnO powders, with and without dye) were compared. The manufacturing and experimental conditions for the photovoltaic measurements were equivalent for all cells. Thus, the different performances could be attributed to the differences presented by nanoparticles, such as dye adsorption capacity, electron-hole pair lifetime, and electron mobility.

### 2.2. ZnO Characterization

X-ray diffraction (XRD) performed on a Bruker D2 diffractometer (Billerica, MA, USA), with Cu-Ka radiation in the 5° < 2θ < 75° range, with steps of 0.05° every 1 s, was carried out for phase identification. Crystal size was calculated using the Scherrer equation for the best-defined peaks.

A Jeol JSM6300 (Akishima, Japan) scanning electron microscopy (SEM) with an energy-dispersive X-ray spectroscopy (EDS) by Jeol was used for morphological analysis and composition analysis. A Brunauer, Emmett and Teller (BET) surface area analysis was performed with a gas adsorption instrument Autosorb Quanta chrome model NOVA 1000 (, Boynton beach, FL, USA). A diffuse reflectance spectroscopy with a Kubelka–Munk reemission function was used to determine the optical bandgap values in an Ocean Optics model DH-20000 (Bryan Dairy Road, Largo, FL, USA) equipped with an integrating sphere Ocean Optics model ISP-REF 2.2.

### 2.3. Solar Cells Assembly and Performance Measurement

Dye-sensitized and dye-free solar cells were assembled with ZnO-B and ZnO-C.

To prepare the glass substrates, FTO glass (Sigma-Aldrich - 7Ω/sq) was cut to the dimensions of 30 × 15 mm. The counter electrode FTO glass was drilled with a 1 mm diamond drill in the geometric center. Subsequently, the assembly of the solar cells took place in the following steps.

FTO glass cleaning was performed in an ultrasound bath for 10 min with 1% v/v detergent, 10 min with distilled water, and 10 min with ethanol, in this order. The glasses were then dried with a jet of compressed air.

The deposition of porous oxide films in the photoanode started from a slurry with the oxides produced using approximately 0.3 g of oxide, 1 mL of ethanol, 18 μL of ethylene glycol, and 15 μL of Triton X-100. The deposition of a 1 cm^2^ layer of TiO_2_ or ZnO was made by a doctor blade producing 90 µm layers after drying. The semiconductor layer was dried for approximately 5 min and then sintered in a muffle furnace at 450 °C for 30 min.

Impregnation with the dye molecules was performed by leaving the glasses with the oxide layers dipped in a 0.2 mM alcohol solution of the commercial dye N749 BLACK DYE for 24 h. After the immersion period, they were washed with ethanol and dried at room temperature. The dye amount incorporated in both cases could not be determined.

After drying the perforated FTO glass, the process for depositing the catalyst and sealing the cells started. The glass surface was coated with graphite and carefully placed on the cell with a 50 micron thick polymeric spacer to form the counter electrode. The sealing of the cells was done with the aid of binder clips to facilitate the application of the epoxy adhesive along the contact edges.

The electrolyte used for deposition was the Dyesol brand’s EL-HPE High Performance Electrolyte. It was deposited using a silicone nozzle syringe, which allows a good seal on the 1 mm hole in the counter electrode and vacuum formation inside the cell. Later the hole was sealed.

Photocurrent–voltage I-V measurements were performed using a Keithley 2650 (Cleveland, OH, USA) Source Measure Unit (SMU) with 4 channels interfaced, and a Hamamatsu L8029 Xenon lamp (Shizuoka, Japan) as the source. All measurements were performed under a carefully controlled sample temperature and in complete darkness.

## 3. Results

### 3.1. ZnO Characterization

**Figure 1:**
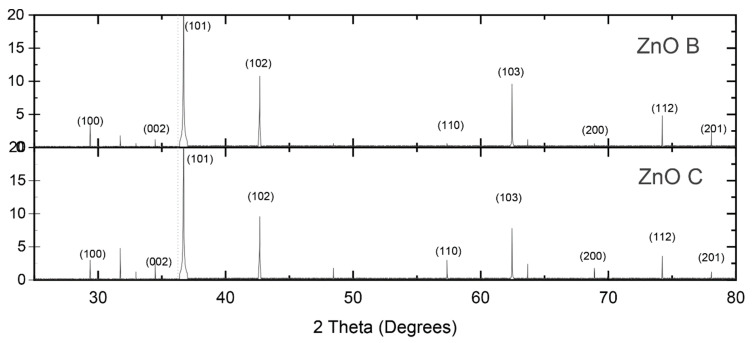
Diffractogram of ZnO samples showing zincite crystallization.

The calcination step in the biomimetic synthesis served for the removal of the organic material and crystallization of the oxides. By calcining at 600 °C, complete removal of the organic material was obtained without a large growth of the crystals [2]. X-ray diffraction analysis revealed the presence of only zincite phase in both commercial and biomimetic ZnO powders (Figure 1). The difference of the spectra occurred in the intensities and widths of the peaks that showed smaller crystallite sizes for biomimetic powders. The crystallite size measurements are presented in Table 2, along with the surface areas calculated by the BET. Photocurrent–voltage I-V measurements were performed using a Keithley 2400 Standard SMU.

For a good DSSC performance, the semiconductor must present high values of both electron diffusion coefficient (D) and electron recombination lifetime (τ). Nakade et al. [15] found that for TiO_2_ nanoparticles, D increases and τ decreases with increasing crystallite size up to 32 nm, and therefore exists at an optimal crystallite size for this value. In Table 2, the results obtained for both ZnO powder types are presented.

It was expected that for a larger area, a greater adsorption tendency of dye molecules would increase performance. Therefore, the biomimetic powder presented promising results regarding the crystallite size and surface area aspects, since it had a small crystallite size (10.2 nm) and a larger surface area (20.6 m^2^/g) compared to commercial powder (8.9 m^2^/g).

**Figure 2:**
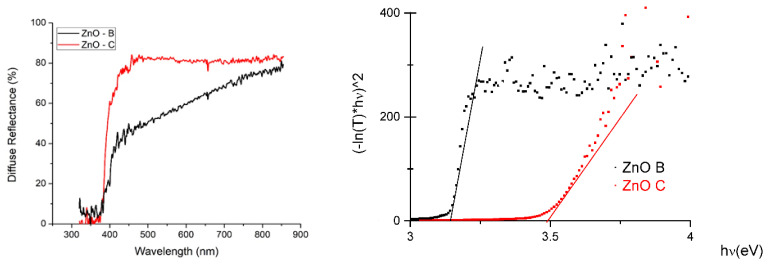
Diffuse ZnO reflectance plot (commercial and biomimetic). Tauc plot is shown on the right.

The results of diffuse reflectance (Figure 2) showed a different behavior for the biomimetic and commercial powder, since the commercial powder presented a well-defined absorption jump in the violet region. For the biomimetic powder, the jump in absorption occurred apparently in two steps. It is believed that due to the higher density of crystalline defects present in the biomimetic powders, they have more acceptor and/or donor levels within their bandgap, causing larger wavelengths to be absorbed by the material as well. The smaller crystallite size verified by the XRD is indicative of more grain boundaries and therefore more defects in the biomimetic samples. Furthermore, according to [2], in biomimetic ZnO powders, there may be the presence of impurities, such as sulfur and/or phosphorus, that can contribute to the formation of point defects that contribute to the modification of the bandgap. From the diffuse reflectance data, the bandgap of the samples was calculated with the Kubelka–Munk remission function. From the results presented in Table 2, it can be seen that the biomimetic powder has a smaller bandgap (3.18 eV) than the analog commercial powder (3.49 eV), indicating that the biomimetic ZnO can absorb a broader range of wavelengths of visible light.

**Figure 3:**
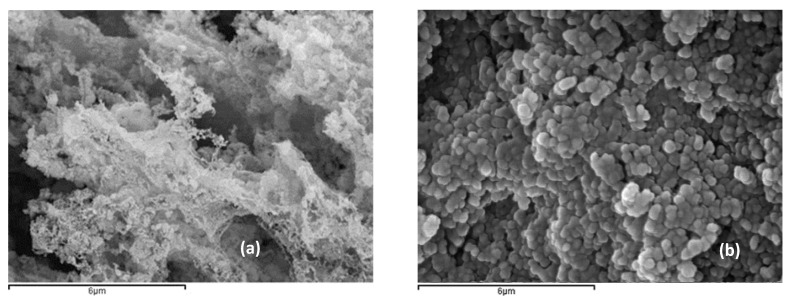
SEM images showing the fragile fiber structures of the biomimetic particles and the equiaxial aspect of the commercial particles. (**a**) biomimetic ZnO, (**b**) commercial ZnO.

SEM analysis (Figure 3) of the biomimetic particles revealed the formation of a very fragile tubular fibrous structure, as well as those reported by other authors [15]. The lamellar appearance of the zincite crystals that grow in the tube walls made the fibrous structure collapse easily. The structure presented by the biomimetic powder favored a higher adsorption of dye molecules because they had high porosity (verified by the BET), small crystallite sizes (checked from XRD), and, if the tubular structure was maintained, it was able to trap dye molecules both on their inner and external walls. The crystallite sizes observed on the SEM images were different (normal due to the localized measurement) from the BET values, but maintained smaller relative sizes for the biomimetic powder.

### 3.2. Solar Cells Characterization

**Figure 4:**
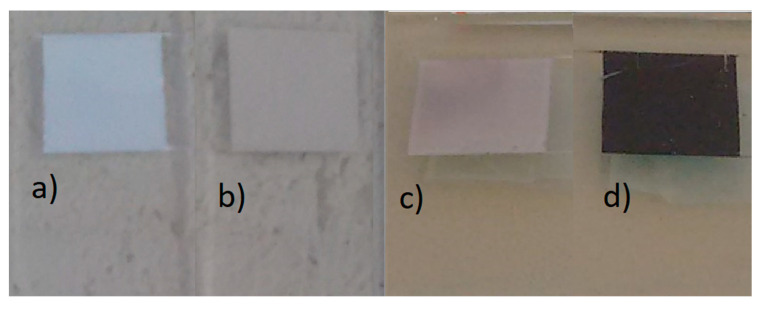
Photographs of FTO glass substrates coated with the semiconductor: (**a**) ZnO-C without dye; (**b**) ZnO-B without dye; (**c**) ZnO-C with dye; (**d**) ZnO-B with dye.

The cells were later assembled with the different powders (biomimetic and commercial) and with or without dye. In Figure 4, the important difference in color and opacity is evident. It is clear that the biomimetic ZnO cell (Figure 4d) became much darker with the presence of the dye, meaning that the porosity of the powders was adequate for the adsorption of large amounts of dye molecules.

**Figure 5:**
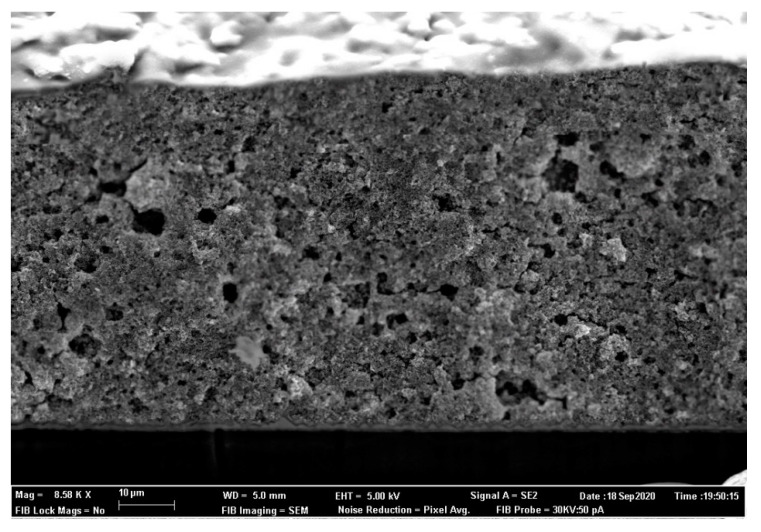
FESEM image of the fibbed cross-section of the ZnO-B sample where the approximately 60 µm thick layer is observed over the FTO layer.

In Figure 5, a cross-section of the doctor-bladed ZnO-B layer is shown for reference, clearly showing its porous microstructure well-appreciated over the FTO conducting layer.

**Figure 6:**
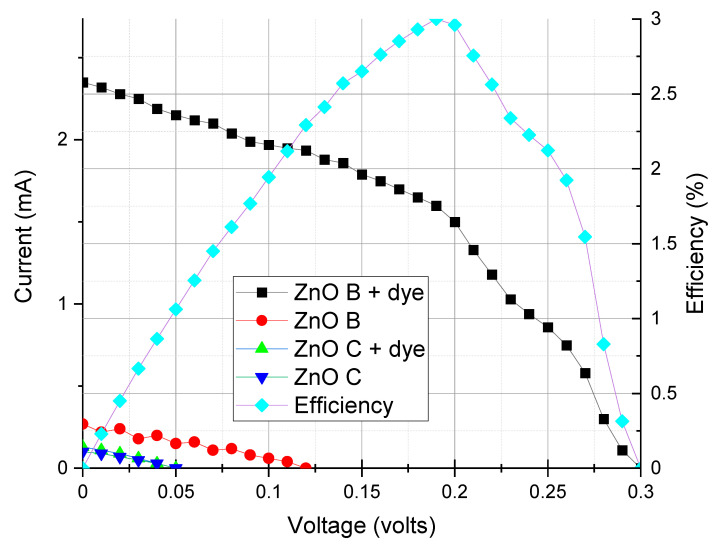
I-V curves for ZnO based DSSC cells.

Analyzing the I-V curves (Figure 6) of the different solar cells, we noticed that the dye potentiated the generation of energy for both biomimetic and commercial powders. A much more pronounced gain in efficiency was noted for the cell prepared with the biomimetic ZnO. The results, therefore, indicated that the presence of the N749 dye acted differently for the biomimetic and commercial ZnO, demonstrating to be more suitable for use with biomimetic ZnO. The efficiency curve in Figure 5 shows an interesting performance of the biomimetic ZnO with dye.

The results presented by the biomimetic ZnO cells are promising, and we believe that the crystalline characteristics, such as low crystallite size, high surface area, and presence of light absorption defects presented by ZnO-B, are close to the ideal ones for the construction of ZnO DSSCs.

## 4. Discussion

The experimental results obtained for bio-synthesized ZnO were clearly superior to commercial powders obtained (as is the case in our commercial ZnO powder) from a chemical synthesis followed by mechanical size reduction. The crystalline quality (lower concentration of defects) observed on the commercial sample did not provide the expected [12] PV performance. This was magnified with dye presence. The explanation considered was based on the progressive increase of diffused reflectance observed in Figure 2 for ZnO-B, which might provide more efficient charge mobility.

On the other hand, the greener production of bio-synthesized powders added a powerful incentive for building integrated solar cells printed directly from bio-templates for enhanced power conversion efficiency on inkjet-printed cells with more advanced designs [16].

## 5. Conclusions

DSSCs were produced with biomimetic and commercial ZnO with and without dyes. The performance of the cells with biomimetic ZnO powders was quite superior to the performance of the commercial ZnO cells. The presence of the dye greatly increased the open-circuit voltage (VOC) and short-circuit current (JSC) values for both ZnO cells. The biomimetic synthesis route proved to be a simple route capable of producing powders with small crystallite sizes (10.2 nm), high surface area (20.6 m^2^/g), and smaller bandgaps than its highly crystalline commercial analogs. In this context, the photovoltaic characterization of the cell assembled with biomimetic ZnO presented distinctly better results than the cells assembled with commercial ZnO, thus showing the great potential of the use of biomimetic ZnO for the production of DSSCs.

## Figures and Tables

**Table 1:** Naming convention for powders under study.

ZnO	Biomimetic	Commercial
Naming convention	ZnO-B	ZnO-C

**Table 2:** Crystallite sizes measured by the Scherrer equation and surface areas measured by the BET.

ZnO type	ZnO-B	ZnO-C
Crystallite size (nm)	10.2	75.5
Surface area (m^2^/g)	20.6	8.9
Bandgap (eV)	3.18	3.49
